# Anchoring the CerEla1.0 Genome Assembly to Red Deer (*Cervus elaphus*) and Cattle (*Bos taurus*) Chromosomes and Specification of Evolutionary Chromosome Rearrangements in Cervidae

**DOI:** 10.3390/ani11092614

**Published:** 2021-09-06

**Authors:** Miluse Vozdova, Svatava Kubickova, Halina Cernohorska, Jan Fröhlich, Jiri Rubes

**Affiliations:** Department of Genetics and Reproductive Biotechnologies, Central European Institute of Technology—Veterinary Research Institute, 62100 Brno, Czech Republic; kubickova@vri.cz (S.K.); cernohorska@vri.cz (H.C.); frohlich@vri.cz (J.F.); rubes@vri.cz (J.R.)

**Keywords:** BAC mapping, comparative cytogenetics, chromosome fission, chromosome fusion, FISH, genome assembly, karyotype

## Abstract

**Simple Summary:**

The red deer (*Cervus elaphus*) de novo genome assembly (CerEla1.0) has provided a great resource for genetic studies in various deer species. In this study, we used gene order comparisons between *C. elaphus* CerEla1.0 and *B. taurus* ARS-UCD1.2 genome assemblies and fluorescence in situ hybridization (FISH) with bovine BAC probes to verify the red deer-bovine chromosome relationships and anchor the CerEla1.0 C-scaffolds to karyotypes of both species. We showed the homology between bovine and deer chromosomes and determined the centromere-telomere orientation of the CerEla1.0 C-scaffolds. Using a set of BAC probes, we were able to narrow the positions of evolutionary chromosome breakpoints defining the family Cervidae. In addition, we revealed several errors in the current CerEla1.0 genome assembly. Finally, we expanded our analysis to other Cervidae and confirmed the locations of the cervid evolutionary fissions and orientation of the fused chromosomes in eight cervid species. Our results can serve as a basis for necessary improvements of the red deer genome assembly and provide support to other genetic studies in Cervidae.

**Abstract:**

The family Cervidae groups a range of species with an increasing economic significance. Their karyotypes share 35 evolutionary conserved chromosomal segments with cattle (*Bos taurus*). Recent publication of the annotated red deer (*Cervus elaphus*) whole genome assembly (CerEla1.0) has provided a basis for advanced genetic studies. In this study, we compared the red deer CerEla1.0 and bovine ARS-UCD1.2 genome assembly and used fluorescence in situ hybridization with bovine BAC probes to verify the homology between bovine and deer chromosomes, determined the centromere-telomere orientation of the CerEla1.0 C-scaffolds and specified positions of the cervid evolutionary chromosome breakpoints. In addition, we revealed several incongruences between the current deer and bovine genome assemblies that were shown to be caused by errors in the CerEla1.0 assembly. Finally, we verified the centromere-to-centromere orientation of evolutionarily fused chromosomes in seven additional deer species, giving a support to previous studies on their chromosome evolution.

## 1. Introduction

The family Cervidae (Ruminantia) groups more than fifty extant deer species, including species with growing economic importance. Deer species can be divided into three subfamilies: Cervinae, Capreolinae and Hydropotinae [1] and show a great karyotype diversity reflecting chromosome evolution of the taxon. The diploid chromosome numbers range from 2n = 6 in the female Indian muntjac (*Muntiacus vaginalis*) to 2n = 70 in several species of Capreolinae [2,3,4]. The 2n = 70 karyotypes of *Hydropotes inermis* and *Mazama gouzoubira*, involving 68 acrocentric autosomes, an acrocentric X and a small submetacentric Y, most probably represent an ancestral cervid karyotype [4] which evolved from the hypothetical ancestral pecoran karyotype (2n = 58) by six chromosome fissions [5]. 

Comparative cytogenetic studies revealing interspecies chromosome homologies and tracking of evolutionary karyotype rearrangements have been still scarce in Cervidae, with the exception of Muntjacini. The published studies were based mostly on standard banding methods [6,7,8] or on fluorescence in situ hybridisation (FISH) using whole chromosome painting probes [5,9,10,11,12,13]. The known data show that the most common mechanism of karyotype evolution in Cervidae is represented by Robertsonian (centric) fusions [4,7], whereas tandem fusions were described as the major evolutionary karyotype shaping factor in Muntiacini [9,11]. On the other hand, fissions of several ancestral pecoran chromosomes conserved in *Bos taurus* (BTA, 2n = 60) as BTA1, 2, 5, 6, 8, 9 and intrachromosomal rearrangements of the BTA1 orthologue and the X chromosome were also detected in Cervidae using bovine BAC (Bacterial Artificial Chromosome) probes [13,14]. 

However, the recent rapid development of high throughput molecular methods, namely whole genome sequencing, has brought new resources for comparative phylogenetic studies. At the level of chromosomes and their parts, an analysis of the next generation sequencing data can enable a precise determination of evolutionary chromosome breakpoints and allow a detection of small or intrachromosomal rearrangements that cannot be visualized by conventional cytogenetics or FISH with whole chromosome painting probes. In ruminants, cryptic interspecies chromosome differences as small as 3.3 Mb were identified in cattle and sheep using an in silico comparative bioinformatic approach [15]. This indicates that the use of sensitive methods can bring interesting discoveries even in seemingly well-described taxa.

Unfortunately, this approach is only limited to species with completely sequenced and well-assembled genomes. Regarding Cervidae, whole genome assembly divided to chromosome-scale scaffolds (C-scaffolds) and including basic gene annotation is available only for the red deer (*Cervus elaphus*, CEL, 2n = 68) [16]. The CerEla 1.0 assembly available in the NCBI database has a total length of 3438.62 Mb and a total ungapped length 1960.83 Mb. It includes 406,637 contigs, 11,479 scaffolds and 35 chromosome-scale scaffolds (C-scaffolds) (https://www.ncbi.nlm.nih.gov/assembly/GCA_002197005.1/#/st accessed on 5 November 2020). The C-scaffolds in the CerEla1.0 genome assembly currently available in the NCBI database are arranged in accordance with the red deer genetic linkage map [17]. As a result, their order does not comply with the physical chromosome length and the chromosome order and centromere-telomere orientation in the red-deer karyotype. 

Generally, the use of other methods, i.e. BAC FISH mapping, is recommended to verify the newly established genome assemblies and physically anchor them to to chromosomes, thus upgrading them to a chromosome level [18,19,20,21]. In this study, we used comparisons with cattle (*B. taurus*), a closely related species used as model for comparative studies among Cetartiodactyla, with a range of available BACs and, above all, a well established whole genome sequence that served as a reference sequence for the CerEla1.0 assembly establishment [16]. We paired the 34 deer chromosome-scale scaffolds of the *C. elaphus* (CerEla1.0) genome assembly with bovine chromosomes by comparison of the gene annotation of the *C. elaphus* (CerEla1.0) and *B. taurus* (ARS-UCD1.2) assemblies available in the NCBI database. We selected bovine BACs for a construction of FISH probes that we used to anchor the CerEla1.0 C-scaffolds to *C. elaphus* karyotype, to compare the centromere-telomere orientation of the deer and bovine chromosomes and to analyse cervid evolutionary chromosome rearrangements. Using this approach, we revealed and corrected several incongruences between the CerEla1.0 and ARS-UCD1.2 genome assemblies, specified the orientation of the *C. elaphus* C-scaffolds and adjusted the predicted positions of evolutionary breakpoints characteristic for the cervid lineage. Using BAC-FISH mapping, we verified the breakpoints positions in a total of eight karyotypically different cervid species from subfamilies Cervinae and Capreolinae and specified the centromere-telomere orientation of their evolutionarily rearranged chromosomes.

## 2. Materials and Methods

### 2.1. Samples and Karyotype Analysis

Samples of whole peripheral blood of cattle (*Bos taurus*) and eight deer species including the red deer (*C. elaphus*) were obtained from captive born animals held in the Prague zoological garden and/or in deer enclosures in Bila Lhota and Frycovice (Czech Republic). The analysed species are listed in Table 1. Taxonomic nomenclature published by Groves and Grubb (2011) was used in this study [22]. 

Peripheral blood lymphocytes were cultured, harvested and fixed according to the previously described protocols [23]. Metaphase chromosome spreads for the karyotype and FISH analysis were prepared according to the procedures described previously [24]. GTG-banded karyotypes of *B. taurus* and *C. elaphus* were prepared using the standard trypsin/Giemsa method [25]. The karyotype of *C. elaphus* was arranged in accordance with the previously published deer karyotypes [13,26].

### 2.2. Chromosome Orthology and Breakpoint Site Prediction

Orthology between the red deer and bovine chromosomes was assessed by a comparison of *B. taurus* ARS-UCD1.2 and *C. elaphus hippelaphus* CerEla1.0 annotated genome assemblies available in the NCBI database (Accessed on 15 May 2020). Predicted locations of protein coding genes in the CerEla1.0 genome assembly (https://www.ncbi.nlm.nih.gov/genome/browse/#!/proteins/10790/321837%7CCervus%20elaphus%20hippelaphus/ accessed on 15 May 2020) were compared with positions of the corresponding genes in the bovine genome (https://www.ncbi.nlm.nih.gov/gene/advanced accessed on 15 May 2020). Briefly, we selected predicted protein coding genes separated by a distance of approximately 5 Mb along the length of the *C. elaphus* CerEla1.0 C-scaffolds and searched for their positions in the bovine ARS-UCD1.2 genome assembly. The 5 Mb distance was chosen to enable a reliable distinguishing of the mutual positions of BAC probes mapping to these regions when any incongruences would need to be solved by a dual colour BAC-FISH. To specify the breakpoints of the evolutionary chromosome fissions of ancestral chromosomes corresponding to bovine BTA1, 2, 5, 6, 8 and 9 [13,14], we predicted the putative ancestral breakpoint sites on the basis of the flanking gene positions in the deer and bovine genome assembly. The real positions of the evolutionary breakpoints were narrowed using a set of BAC probes and verified in all deer species available for this study.

### 2.3. FISH Probes

BAC clones specific to proximal and distal chromosome regions, to regions flanking the predicted evolutionary breakpoint sites in Cervidae, and to regions showing incongruences between the deer and bovine genome assembly were selected from the CHORI-240 bovine BAC library (BACPAC Genomics, Emeryville, CA, USA) on the basis of their location along the bovine chromosomes in the ARS-UCD1.2 genome assembly. The chromosome positions of cervid evolutionary breakpoints were further narrowed using additional BAC clones located in neighbouring positions. The BAC clones used in this study are listed in Supplementary Tables S1–S3. The BAC DNA was isolated using Wizard Plus SV Minipreps DNA Purification System (Promega, Madison, WI, USA), labelled with Green-dUTP (Abbott, Abbott Park, IL, USA), biotin-16-dUTP (Roche, Mannheim, Germany), or digoxigenin-11-dUTP (Roche) using BioPrime Array CGH Genomic Labeling Module (Invitrogen, Carlsbad, CA, USA) and used for FISH.

### 2.4. FISH

A hybridization mixture containing 50% formamide, 2 × SSC, 10% dextran sulfate, 0.7 μg salmon sperm, 1.3 μg Bovine Hybloc DNA (Applied Genetics Laboratories, Melbourne, FL, USA) and 200 ng of the labeled DNA probe was prepared. Ten μL of the mixture were denatured at 75 °C for 10 min, preannealed at 37 °C for at least 30 min, and applied on slides with metaphase chromosomes denatured by 0.07 M NaOH as previously described [27]. After hybridization in a humid chamber at 37 °C overnight, the slides were washed in 0.7 × SSC at 72 °C for 2 min. The BAC probes labeled with biotin-16-dUTP or digoxigenin-11-dUTP were detected with Avidin-Cy3 (Amersham Pharmacia Biotech, Piscataway, NJ, USA), Streptavidine-Cy5 (Invitrogen/Molecular Probes, Camarillo, CA, USA) and antidigoxigenin-rhodamine (Roche) according to manufacturers’ instructions. If we used a combination of two probes labelled/detected by the same fluorochrome for the same chromosome, we performed two rounds of FISH, so that the position of each probe could be reliably determined. The slides were mounted in Vectashield mounting medium containing 1.5 mg DAPI (Vector Laboratories) and analysed using Zeiss Axio Imager.Z2 fluorescence microscope (Carl Zeiss Microimaging GmbH, Jena Germany) equipped with appropriate fluorescent filters and the Metafer Slide Scanning System (MetaSystems, Altlussheim, Germany). Images of well-spread metaphase cells were captured by CoolCube CCD camera (MetaSystems) and analysed using Isis3 software (MetaSystems). The reliability of the BAC probes was confirmed by their hybridization on bovine chromosomes prior to FISH in deer.

## 3. Results

**Figure 1:**
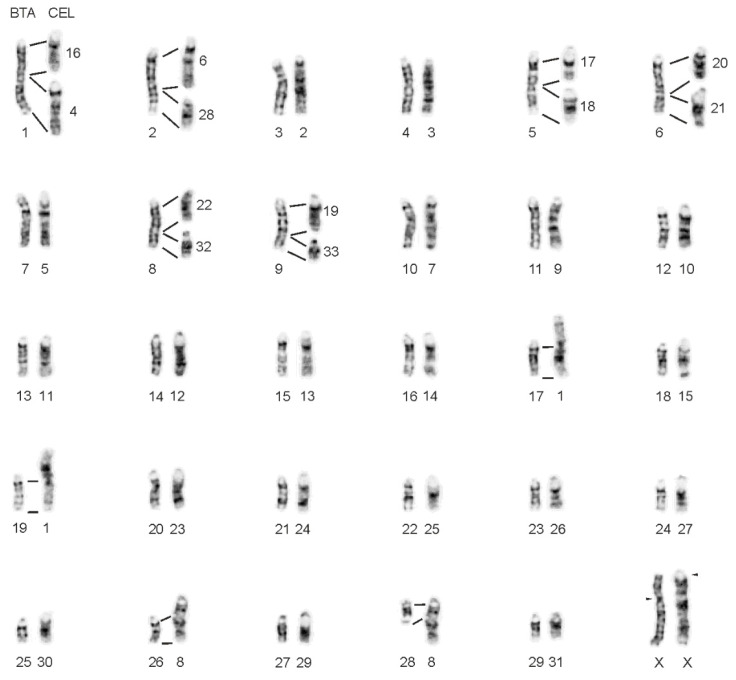
The orthology between the G-banded chromosomes of cattle (*B. taurus*, BTA) and red deer (*C. elaphus*, CEL). Arrowheads indicate the position of centromeres on the X chromosomes.

**Figure 2:**
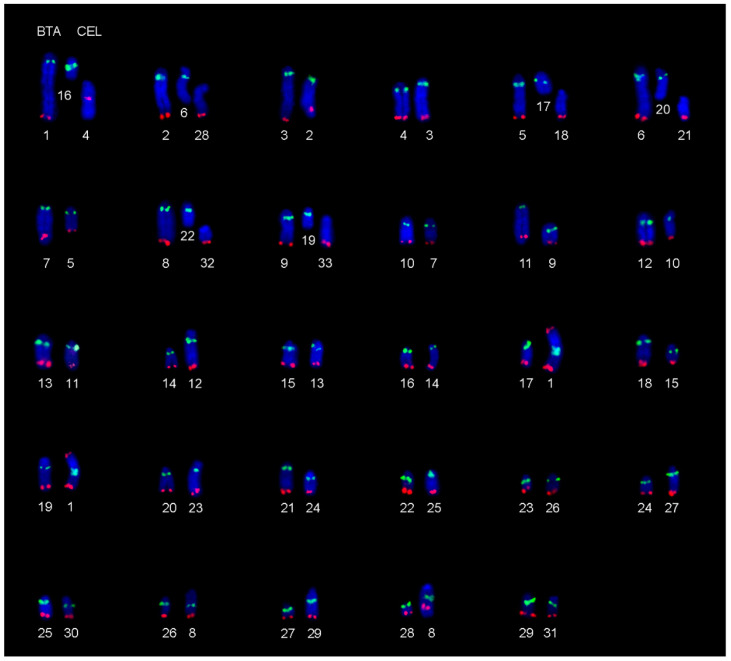
Centromere-telomere orientation of orthologous bovine (*B. taurus*, BTA) and red deer (*C. elaphus*, CEL) chromosomes confirmed by BAC-FISH. Green signal—proximal BAC probe; red signal—distal BAC probe.

Comparing chromosomal positions of the predicted genes annotated to the CerEla1.0 C-scaffolds with their locations in the bovine ARS-UCD1.2 genome assembly, we assigned all red deer C-scaffolds to their bovine orthologues (Supplementary Table S4). Then we verified the deer-bovine chromosome orthology by BAC-FISH, which also enabled reliable physical anchoring of CerEla1.0 C-scaffolds to *C. elaphus* karyotype. Using BAC probes, we observed identical physical centromere-telomere orientation of orthologous red deer and bovine chromosomes. However, the orientation of CerEla1.0 C-scaffolds 2, 6, 8, 11, 12, 16, and 22 in the NCBI database was found to be reversed, and the deer chromosome CEL4 was found rearranged, when compared with the corresponding CerEla1.0 C-scaffold 19. The orthology between the G-banded red deer and cattle karyotypes are displayed in Figure 1 and Supplementary Figure S1. The relationships among the CerEla1.0 C-scaffolds and the red deer and cattle chromosomes are summarized in Table 2. The comparative FISH results in cattle and the red deer are documented in Figure 2. Karyotypes of the additional studied cervid species with indicated homologies with *B. taurus* are displayed in Supplementary Figure S2.

**Figure 3:**
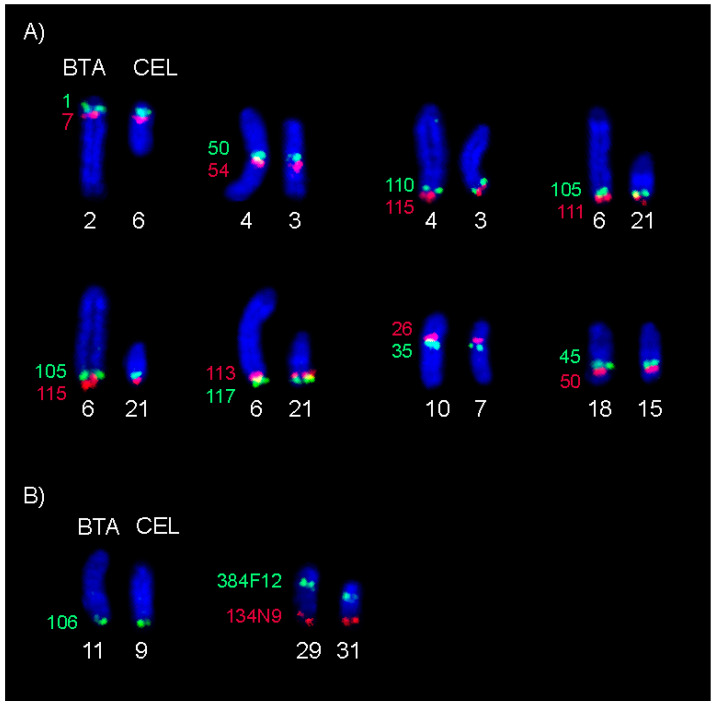
BAC-FISH mapping of the selected regions showing different gene order in CerEla1.0 and ARS-UCD1.2 genome assembly. Numbers indicate the BAC positions (Mb) on individual BTA chromosomes. (**A**,**B**) Identical signal order on orthologous bovine and red deer chromosomes. (**B**) Signal of the BAC probe CH240-134N9 on BTA29 and CEL31 instead of BTA11 and CEL9.

Minor differences in the gene order revealed between CerEla1.0 C-scaffolds 4, 5, 6, 11, 12, 18, 19, 23, 27, 33 and X and their bovine orthologues in the ARS-UCD1.2 genome assembly are highlighted in Supplementary Table S4. We clarified the incongruences in eight of these regions on six red deer chromosomes, i.e., where the order of the BAC probes mapping to the incongruent regions was clearly visible. We observed an identical order of the BAC-FISH signals on cattle and red deer in all of the regions (Figure 3). Interesting results were obtained using the BAC probe CH240-134N9 targeted to the incongruent proximal region of the C-scaffold 11 corresponding to the distal part (82.9 Mb) of BTA11 according to CerEla1.0 and ARS-UCD1.2 comparisons, and to orthologous *C. elaphus* chromosome CEL9. Instead of BTA11 and CEL9, this probe hybridized to a distal part of other chromosome in both cattle and red deer. This chromosome was subsequently identified as BTA29, and CEL31, respectively, by FISH with the BAC probe CH240-384F12 specific to the proximal part (5.8 Mb) of BTA29 orthologous to CEL31 (Figure 3B).

Regarding the X chromosome, we found that the sequences spanning 1–86 Mb of the CerEla1.0 X chromosome C-scaffold copy the gene order of the bovine X chromosome. However, a different order of the evolutionary conserved X chromosome segments was previously reported in studies using BAC-FISH in Cervidae [13,28]. 

**Figure 4:**
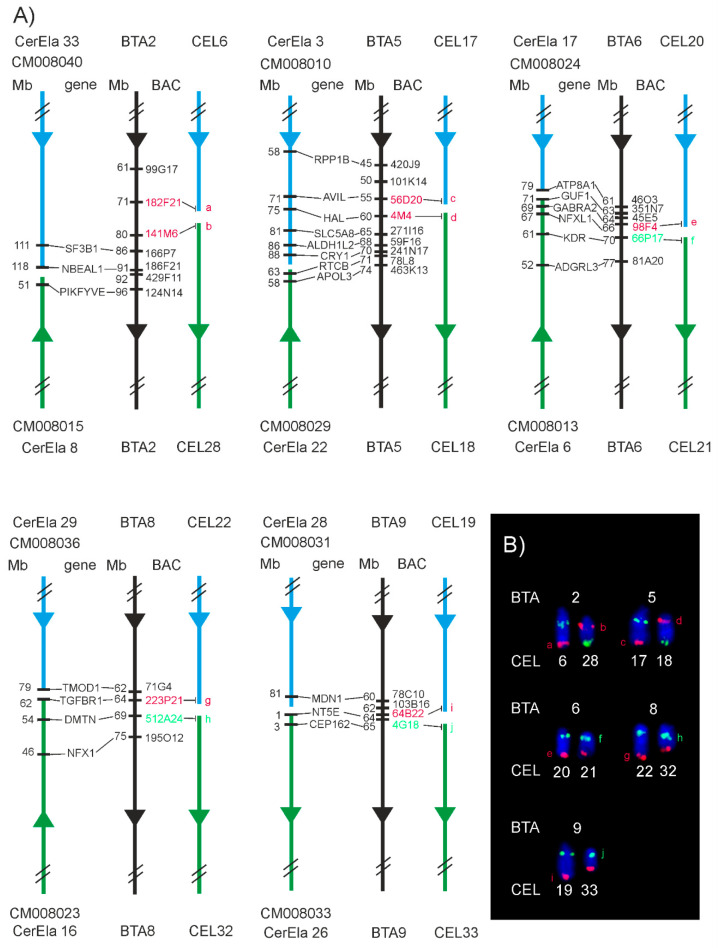
Evolutionary breakpoints on BTA2, 5, 6, 8 and 9 orthologues. (**A**) Schematic presentation of the CerEla1.0 C-scaffolds and BTA and CEL chromosomes with indicated positions of BAC clones used for the verification of the breakpoint positions. The BAC gene content and the position of the genes on the CerEla1.0 C-scaffolds is also shown. Notice differences in the assumed breakpoint positions on the CerEla1.0 C-scaffolds and the positions of breakpoints detected by FISH on the red deer chromosomes. (**B**) BAC-FISH signals at proximal, breakpoint and distal positions on *C. elaphus* chromosomes orthologous to BTA2, 5, 6, 8 and 9. Positions of selected BAC probes indicating the approximate evolutionary breakpoints are marked by letters (a–j): a—182F21, b—141M6, c—56D20, d—4M4, e—98F4, f—66P17, g—223P21, h—512A24, i—64B22, j—4G18. The unmarked FISH signals correspond to the proximal (green); and distal (red) BAC probes.

**Figure 5:**
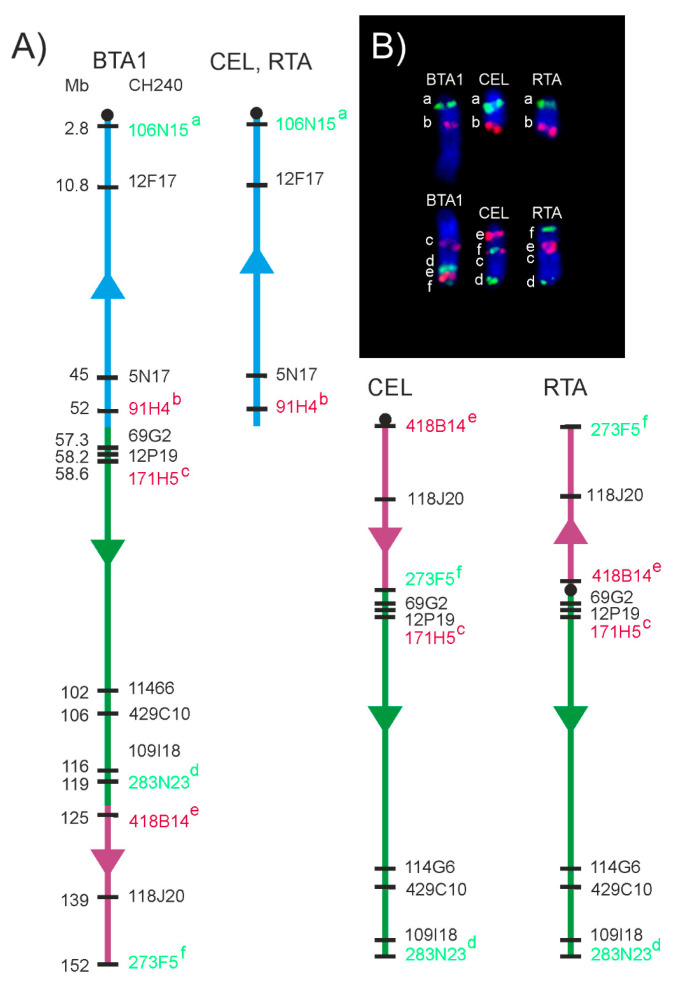
Evolutionary rearrangements of the BTA1 orthologue in Cervidae. (**A**) Schematic presentation. (**B**) BAC-FISH results in *B. taurus* (BTA), *C. elaphus* (CEL) and *R. tarandus* (RTA) using selected individual BAC probes indicated by letters (a–f): a—106N15, b—91H4, c—171H5, d—283N23, e—418B14, f—273F5.

Positions of the evolutionary chromosome breakpoints in chromosomes orthologous to BTA1, 2, 5, 6, 8 and 9 in the cervid ancestor were predicted on the basis of the genes located in the most proximal and distal positions of the corresponding CerEla1.0 C-scaffolds and, thus, flanking the assumed breakpoints. However, we revealed that the real breakpoints were located in a slightly different positions by a physical FISH-mapping with a series of BAC probes distributed along the chromosomes in the proximity of the predicted breakpoints (Figure 4 and Figure 5). The subsequent analysis of the breakpoint positions in additional cervid species showed similar results in all deer species analysed in this study (Supplementary Figure S3).

The evolution of the BTA1 orthologue in the cervid lineage involved an initial fission followed by intrachromosomal rearrangements of one of the newly formed chromosomes. Two differentially rearranged types of the chromosome orthologous to the distal part of the BTA1 were observed in this study: An acrocentric chromosome common to Cervinae and *C. capreolus* and a submetacentric chromosome observed in the remaining Capreolinae in this study (*R. tarandus*, *A. alces* and *O. virginianus*) that was most probably derived from the previous by a pericentric inversion (Figure 5). 

Using CerEla1.0 and ARS-UCD1.2 genome assembly comparisons, the fusion site of the ancestral chromosomes corresponding to BTA17 and BTA19, which roughly represents the position of centromere, was found at 95 Mb of the deer C-scaffold 5 (CEL1) length. The evolutionary fission, giving rise to bovine separated BTA28 and BTA26, was located to 60 Mb of the CerEla1.0 C-scaffold 15 (CEL8).

**Figure 6:**
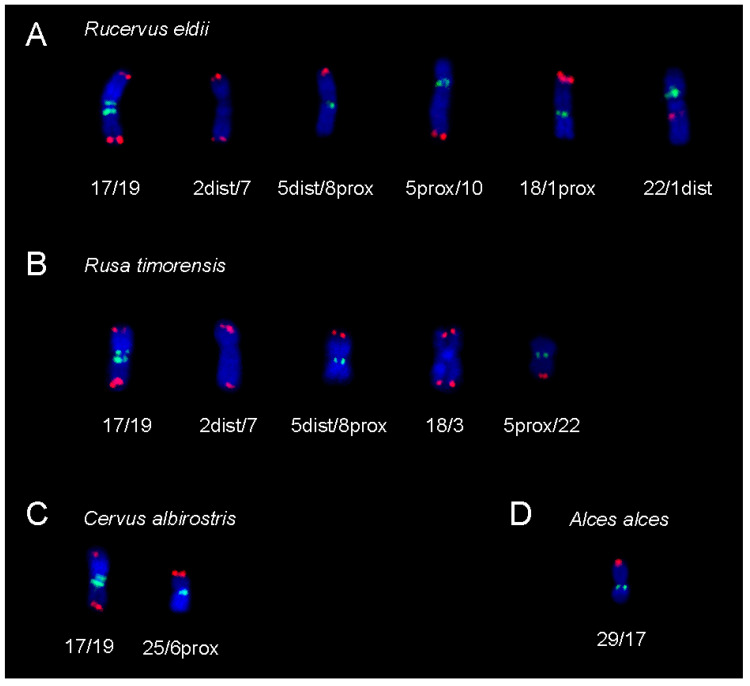
Centromere-telomere orientation of the fused chromosomes in (**A**) *R. eldii*, (**B**) *R. timorensis*, (**C**) *C. albirostris* and (**D**) *A. alces*. Green signal—proximal BAC probe; red signal—distal BAC probe.

Finally, we used the bovine BAC probes to determine the centromere-telomere orientation of their evolutionarily fused chromosomes in seven additional deer species with rearranged karyotypes (Table 1). Except for the tandem fusion of BTA28;26 common to all Cervidae, the rearranged chromosomes were formed by evolutionary centric fusions in all studied species (Figure 6). 

## 4. Discussion

The recent publishing of the *C. elaphus* whole genome assembly (CerEla1.0) [16] brought a great resource for a research in the field of deer evolution, conservation and population genetics. However, the high automation in the genomic assembly construction may lead to errors. A verification and further improvements provided by molecular genetic and cytogenetic approaches are recommended for all newly established genome assemblies [18,19,20,21]. Inter- and intraspecies assembly comparisons supported by FISH enabled the detection and correction of misassembled sequences in genome assemblies of economically important bovid species (cattle, *Bos taurus*, sheep, *Ovis aries* and goat, *Capra hircus*) [29,30]. The combination of bioinformatic comparisons and BAC-FISH allowed identification of cryptic divergences between cattle and goat [15]. Using universal BAC sets, multiple scaffolds can be anchored to chromosomes of various species, as it was shown in birds [20].

In this study, we focused on the verification of chromosome relationships among *C. elaphus* CerEla1.0 and *B. taurus* ARS-UCD1.2 genome assemblies and karyotypes of both species. Using bovine BAC probes, we physically anchored the CerEla1.0 C-scaffolds to *C. elaphus* and *B. taurus* karyotype (Figure 1). Similar approach exploiting BAC-FISH mapping technique was previously successfully used for an integration of cytogenetic landmarks or upgrading draft genome sequences to chromosomal level in other species [20,31]. The C-scaffolds of the CerEla1.0 genome assembly had been constructed according to the reference deer linkage map [17] and the well-established bovine (*B. taurus*) Btau_5.0.1 genome assembly [16]. The order, orientation and schematic length of the C-scaffolds in the NCBI database comply with the deer genetic linkage map [17] but do not correspond with their sequence length in Mb, nor the position of the chromosomes in the red deer karyotype [5,26,32].

To document the results of this study, we arranged the G-banded red deer karyotype with regard to the chromosome morphology, physical lengths and G-banding patterns. Our G-banding and BAC-FISH showed concordant centromere-telomere orientation of the orthologous chromosomes in *C. elaphus* and *B. taurus* karyotypes. In compliance with the published paper on the CerEla1.0 assembly [16], we observed that the CerEla1.0 C-scaffolds 2, 6, 8, 11, 12, 16, 19 and 22 are presented in reversed centromere-telomere orientation in the NCBI database compared with the physical orientation of the red deer and bovine chromosomes.

Comparing the gene order in the CerEla1.0 and ARS-UCD1.2 genome assembly, we observed differences in several CerEla1.0 C-scaffolds. Bana et al. [16] suggested that these red deer genomic regions represent inverted segments. We analysed eight of these regions by BAC-FISH and observed identical BAC probes order in the orthologous bovine and deer chromosomes in all studied regions (Figure 3). Nevertheless, we revealed that the BAC probe CH240-134N9, selected from the position 82.9 Mb of the BTA11 in ARS-UCD1.2 genome assembly, hybridised to a distal part of BTA29 and, correspondingly, to the BTA29 orthologue in the red deer (CEL31). Either the chromosome position of this BAC in the NCBI database is incorrect, or the region covered by this BAC probe in the bovine ARS-UCD1.2 genome assembly and probably the wider region at the start of the CerEla1.0 C-scaffold 11 showing several incongruences with ARS-UCD1.2 (Supplementary Table S4), actually represent sequences of the chromosome BTA29 and CEL31, respectively. The above-mentioned regions of the CerEla1.0 assembly need further thorough revision.

In the published paper on the CerEla1.0 de novo genome assembly, the C-scaffold 33 was supposed to comprise sequences orthologous to parts of chromosomes BTA2 and BTA22 [16]. However, the bovine counterparts of all genes predicted to the CerEla1.0 C-scaffold 33 and selected for the CerEla1.0 - ARS-UCD1.2 comparisons in this study were found on BTA2. 

Our comparisons of the CerEla1.0 C-scaffold X with the bovine chromosome X in ARS-UCD1.2 showed that, despite several smaller discrepancies, the gene order on the CerEla1.0 C-scaffold X corresponds to that on the bovine X chromosome. However, it was previously published that cervid X chromosomes were shaped by complex evolutionary rearrangements, including neocetromere formation, that differentiated them to two distinct types characteristic for Cervinae and Capreolinae [13,28]. With regard to the previously published findings on the X chromosome structure in Cervidae [13,28], the first 86 Mb of the CerEla1.0 X chromosome C-scaffold need to be revised accordingly.

Regarding another evolutionary chromosome changes, it is known that karyotypes of the current deer species derived from the pecoran ancestral karyotype (2n = 58) by fissions of six ancestral chromosomes orthologous to BTA1, 2, 5, 6, 8, 9 [12,13,14,17]. We used BAC probes selected on the basis of CerEla1.0 and ARS-UCD1.2 comparisons to hybridise to positions flanking the predicted evolutionary breakpoints, with the aim to physically verify the breakpoint sites. We revealed that the factual breakpoints differed from those predicted on the basis of CerEla1.0 C-scaffolds by up to 10 Mb, showing that the sequence span of the CerEla1.0 C-scaffolds needs to be properly adjusted. The newly assessed breakpoint locations were proved in all analysed species (four Cervinae and four Capreolinae) in this study.

We also showed that the evolutionary history of the BTA1 orthologue in Cervidae was more complicated than a simple fission and involved also intrachromosomal rearrangements, as was previously suggested [13,16]. The actual evolutionary breakpoint sites on the ancestral BTA1 orthologue, approximated by the set of BAC probes used in this study, diverged from those predicted on the basis of the CerEla1.0 and ARS-UCD1.2 genome assembly comparisons, neither they corresponded to the schematic presentation of the *B. taurus* and *C. elaphus* chromosome differences shown in Bana et al. (2018). Using BAC probes at positions flanking the evolutionary breakpoints, we showed that the primary evolutionary fission of the ancestral chromosome orthologous to BTA1 occurred between 52 and 57 Mb of the BTA1 length. This led to the formation of two neochromosomes with different lengths. The smaller neochromosome orthologous to the proximal part of BTA1 corresponds to CEL16 and CerEla1.0 C-scaffold 31 is present in both Cervinae and Capreolinae. This indicates that this fission of the ancestral BTA1 orthologue together with fissions of BTA2, 5, 6, 8 and 9 orthologues probably represent a defining event of the karyotype evolution of Cervidae. The larger neochromosome orthologous to the distal part of BTA1 then underwent an intrachromosomal rearrangement with a breakpoint between 119 and 125 Mb of the BTA1 length in the common ancestor of *C. capreolus* and the current Cervinae. This rearrangement was followed by a pericentric inversion of the proximal part of the rearranged chromosome during a separate evolution of the lineage leading to *R. tarandus*, *A. alces* and *O. virginianus* (Figure 5). 

Because the BAC-FISH was proved to be an advantageous and sensitive tool for karyotype evolution studies [7,11,13,14,15,33,34,35,36], we used this method for verification of the evolutionary chromosomal rearrangements in Cervidae. The four species of Cervini analysed in this study share the fusion of BTA17;19 previously described on the basis of banding patterns and chromosome painting [7,13,14,37]. Using BAC probes, we proved that the ancestral chromosomes fused by their centromeres. Apart from the BTA17;19, five other centric fusions were proved in *R. eldii*, four in *R. timorensis* and one in *C. albirostris* by BAC-FISH in this study (Figure 6). As for Capreolini, the centric fusion BTA29;17 was confirmed in *A. alces* in this study. The chromosomes involved in the above mentioned fusions were previously identified by FISH with painting probes but their orientation in fused chromosomes could not be further specified by whole chromosome probes [7,13,14].

In general, our analysis of chromosome evolution in the studied cervid species showed that centric fusions probably represented the main evolutionary mechanism shaping their karyotypes. In species analysed in this study, only the chromosome comprising BTA28;26 orthologues (CEL8) was shown to be formed by a tandem (centromere to telomere) fusion. The fact that the BTA28;26 fusion is common to all Cervidae and characteristic for all pecoran species excluding Bovidae [12,13,14,17] suggests that this chromosome probably represents an ancestral chromosome which underwent a fission at the origin of the Bovidae lineage [38]. Centric fusions are generally characteristic for the karyotype evolution in the family Bovidae [38,39]. However, in Cervidae, centric and tandem fusions dominate differentially in individual clades. In the subfamily Cervinae, centric fusions are relatively common in the tribe Cervini but the karyotypes of Muntjacini were diversified by extensive tandem fusions [4,5,11,33,38]. Among Capreolinae, presumed centric fusions occurred in the karyotype evolution of *Ozotoceros bezoarticus*, *Blastocerus dichotomus* and *A. alces* [3,4] (the latter one was proved in this study). On the other hand, both centric and tandem fusions were involved in the karyotype diversification of South-American Capreolinae species of the genus *Mazama* [40,41,42]. This suggests that the karyotype evolution has been driven by different mechanisms in the individual cervid lineages and cytogenetic studies employing BAC-FISH for the detailed differentiation of the evolutionary rearrangements can help in future studies focused on the reconstruction of the cervid phylogeny. 

## 5. Conclusions

In this study, we verified the red deer-cattle chromosome relationships, anchored the CerEla1.0 C-scaffolds to the red deer and cattle karyotype and proved the centromere-telomere orientation of the CerEla1.0 C-scaffolds. We indicated necessary adjustments to the CerEla1.0 genome assembly, including better specification of the sequence span of the chromosomes that underwent evolutionary chromosome fissions. Finally, we proved the location of the cervid evolutionary fissions and orientation of the fused chromosomes in a total of eight cervid species. Our results can serve as a basis for the CerEla1.0 genome assembly improvement, supporting, thus, future research in Cervidae.

## Figures and Tables

**Table 1:** List of analysed species.

Species	Latin Name	Abbrev.	2n	FNa	Bia	X	Fused BTA Orthologues
Red deer	*Cervus elaphus*	CEL	68	68	2	A	17/19
White-lipped deer	*Cervus albirostris*	CAL	66	68	4	A	17/19, 25/6prox
Rusa deer	*Rusa timorensis*	RTI	60	68	10	A	17/19, 5prox/22, 2dist/7, 5dist/8prox, 5prox/22, 18/3
Eld’s deer	*Rucervus eldii*	REL	58	68	12	A	17/19, 2dist/7, 5dist/8prox, 5prox/10, 18/1prox, 22/1dist
Roe deer	*Capreolus capreolus*	CCA	70	68	0	B	
Reindeer	*Rangifer tarandus*	RTA	70	70	2	B	
Moose	*Alces alces*	AAL	68	70	4	B	29/17
White-tailed deer	*Odocoileus virginianus*	OVI	70	70	2	B	

2n—diploid number; FNa—fundamental number of autosomal arms; Bia—number of bi-armed autosomes; BTA—Bos Taurus; A—acrocentric; B—bi-armed. The evolutionary chromosome fusions were detected previously using bovine whole chromosome painting probes [13].

**Table 2:** CerEla1.0 and ARS-UCD1.2 genome assembly comparisons.

Red Deer (CerEla1.0)			CEL Chr	Cattle (ARS-UCD1.2)			Comments *
Pseudochr	INSDC	Size (Mb)		BTA Chr	RefSeq	Size (Mb)	
1	CM0080008.1	104.5	13	15	NC_037342.1	85.01	
2	CM0080009.1	63.26	31	29	NC_037356.1	51.1	Reverse
3	CM0080010.1	88.46	17	5prox (1–70 Mb)	NC_037332.1	120.09	1–55 Mb of BTA5
4	CM0080011.1	81.2	15	18	NC_037345.1	65.82	
5	CM0080012.1	178.03	1	17/19	NC_037344.1	73.17	
					NC_037346.1	63.45	
6	CM0080013.1	73.11	21	6dist (64–118 Mb)	NC_037333.1	117.81	Reverse, 70–118 Mb of BTA6
7	CM0080014.1	66.84	26	23	NC_037350.1	52.5	
8	CM0080015.1	55.92	28	2dist (94–136 Mb)	NC_037329.1	136.23	Reverse, 80–136 Mb of BTA2
9	CM0080016.1	141.95	5	7	NC_037334.1	110.68	
10	CM0080017.1	55.94	30	25	NC_037352.1	42.35	
11	CM0080018.1	140.39	9	11	NC_037338.1	106.98	Reverse
12	CM0080019.1	127.78	7	10	NC_037337.1	103.31	Reverse
13	CM0080020.1	89.79	24	21	NC_037348.1	69.86	
14	CM0080021.1	103.59	14	16	NC_037343.1	81.01	
15	CM0080022.1	125.28	8	28/26	NC_037355.1	45.94	
					NC_037353.1	51.99	
16	CM0080023.1	62.95	32	8dist (64–112 Mb)	NC_037335.1	113.32	Reverse, 69–112 Mb of BTA8
17	CM0080024.1	79.72	20	6prox (1–63 Mb)	NC_037333.1	117.81	1–66 Mb of BTA6
18	CM0080025.1	152.66	3	4	NC_037331.1	120	
19	CM0080026.1	127.24	4	1dist (59–158 Mb)	NC_037328.1	158.53	Rearranged, 57–158 Mb of BTA1
20	CM0080027.1	149.34	2	3	NC_037330.1	121.01	
21	CM0080028.1	107.36	12	14	NC_037341.1	82.4	
22	CM0080029.1	63.92	18	5dist (71–121 Mb)	NC_037332.1	120.09	Reverse, 60–121 Mb of BTA5
23	CM0080030.1	109.47	11	13	NC_037340.1	83.47	
24	CM0080031.1	78.16	25	22	NC_037349.1	60.77	
25	CM0080032.1	96.54	23	20	NC_037347.1	71.97	
26	CM0080033.1	55.1	33	9dist (64–106 Mb)	NC_037336.1	105.45	65–106 Mb of BTA9
27	CM0080034.1	84.64	27	24	NC_037351.1	62.32	
28	CM0080035.1	82.07	19	9prox (1–62 Mb)	NC_037336.1	105.45	1–64 Mb of BTA9
29	CM0080036.1	80.17	22	8prox (1–63 Mb)	NC_037335.1	113.32	1–64 Mb of BTA8
30	CM0080037.1	117.8	10	12	NC_037339.1	87.22	
31	CM0080038.1	75.46	16	1prox (1–58 Mb)	NC_037328.1	158.53	1–51 Mb of BTA1
32	CM0080039.1	60.01	29	27	NC_037354.1	45.61	
33	CM0080040.1	121.43	6	2prox (1–92 Mb)	NC_037329.1	136.23	1–71 Mb of BTA2
X	CM008041.1	181.54		X	NC_037357.1	139.01	
Y	CM008042.1	4.03		-	-	-	

* Reverse—inversed centromere-telomere orientation of the CerEla1.0 sequence; Rearranged—intrachromosomal rearrangement. Factual span on the BTA orthologue verified by BAC-FISH.

## Data Availability

All data is contained within the manuscript and Supplementary Materials. The FISH images are available from the authors upon request.

## Supplementary Materials

The following are available online at https://www.mdpi.com/article/10.3390/ani11092614/s1, Figure S1. The red deer (C. elaphus, CEL) karyotype with indicated orthology with chromosomes of cattle (*B. taurus*, BTA). Figure S2. G-banded karyotypes of (A) *C. albirostris* (CAL), (B) *R. timorensis* (RTI), (C) *R. eldii* (REL), (D) *R. tarandus* (RTA), (E) *A. alces* (AAL) and (F) *O. virginianus* (OVI) with indicated orthology with chromosomes of *Bos taurus* (BTA). The karyotype of RTI was previously published in Frohlich et al. (2017) [13]; the karyotypes of CAL and REL were published in O’Brien et al. (2020) [26]. Figure S3. Evolutionary chromosome breakpoints in BTA2, 5, 6, 8 and 9 orthologues in the analysed cervid species. *C. albirostris* (CAL), *R. timorensis* (RTI), *R. eldii* (REL), *C. capreolus* (CCA), *R. tarandus* (RTA), *A. alces* (AAL) and *O. virginianus* (OVI). Positions of BAC probes indicating the approximate evolutionary breakpoints are marked by letters (a–j): a—182F21, b—141M6, c—56D20, d—4M4, e—98F4, f—66P17, g—223P21, h—512A24, i—64B22, j—4G18. Table S1: BACs for the physical analysis of the cattle-red deer chromosome orthology and centromere-telomere orientation. Table S2: BACs for the analysis of incongruences between CerEla1.0 and ARS-UCD1.2. Table S3: BACs for the specification of evolutionary chromosome breakpoints in Cervidae. Table S4: Comparison of the CerEla1.0 and ARS-UCD1.2 genome assembly with indicated incongruences.
