# Supplementary figures and images for: Anchoring the CerEla1.0 Genome Assembly to Red Deer (Cervus elaphus) and Cattle (Bos taurus) Chromosomes and Specification of Evolutionary Chromosome Rearrangements in Cervidae
Source: Animals (Basel). 2021 Sep 6;11(9):2614. doi: 10.3390/ani11092614 (PMC8465983; doi:10.3390/ani11092614)

CEL BTA

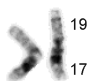

1

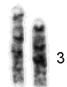

2

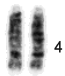

3

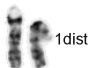

4

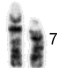

5

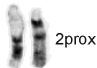

6

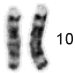

7

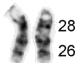

8

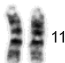

9

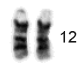

10

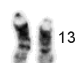

11

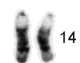

12

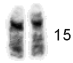

13

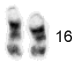

14

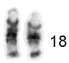

15

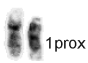

16

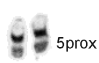

17

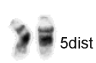

18

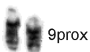

19

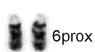

20

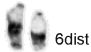

21

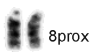

22

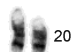

23

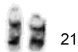

24

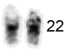

25

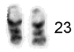

26

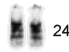

27

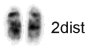

28

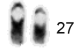

29

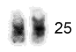

30

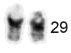

31

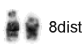

32

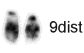

33

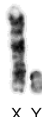

X Y

Supplement: Supplementary file 1 [file animals-11-02614-s001.zip › Suppl Figure S1.pdf]

A

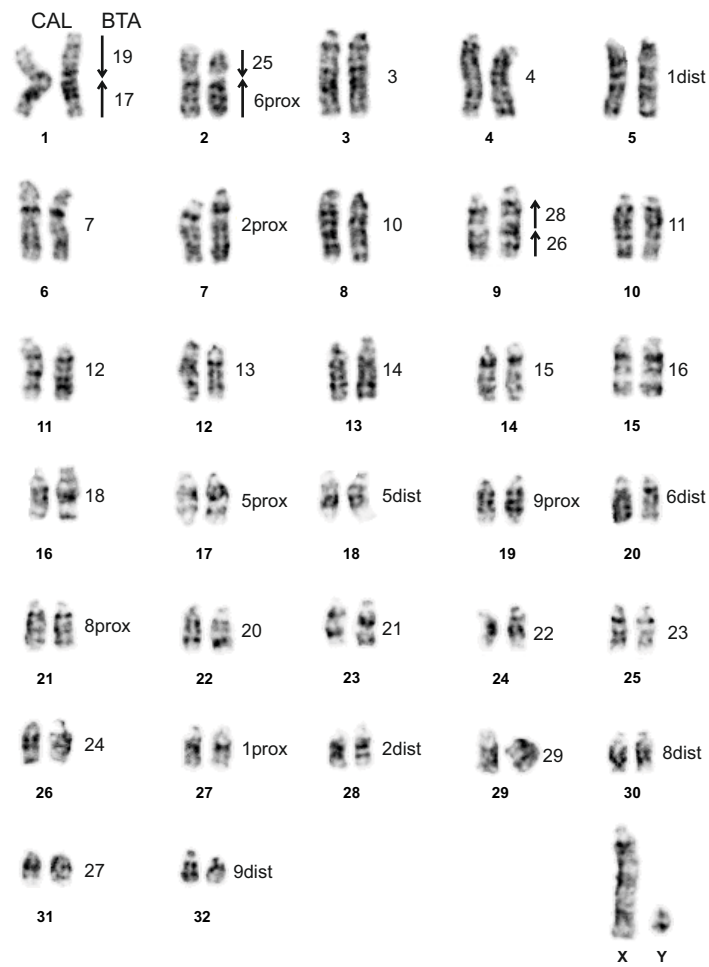

B

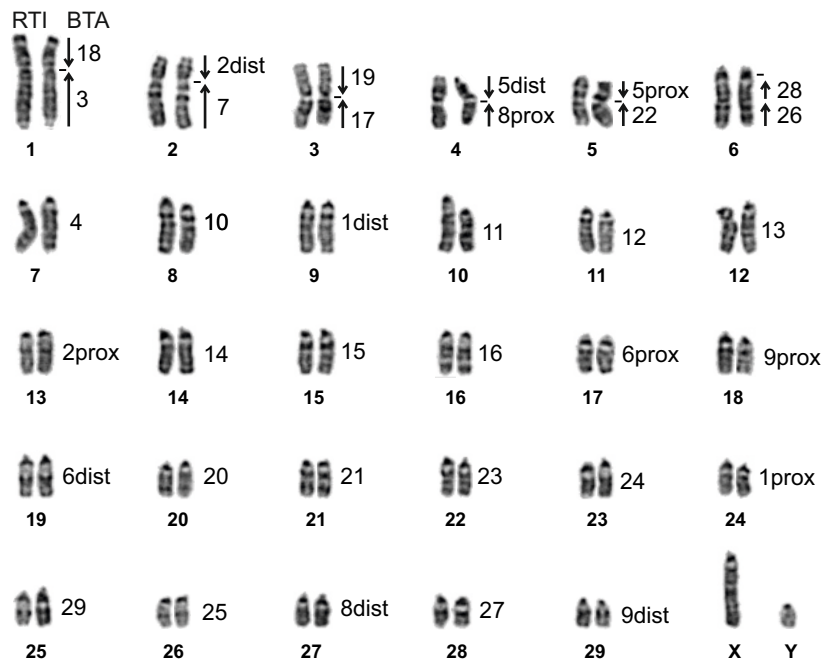

C

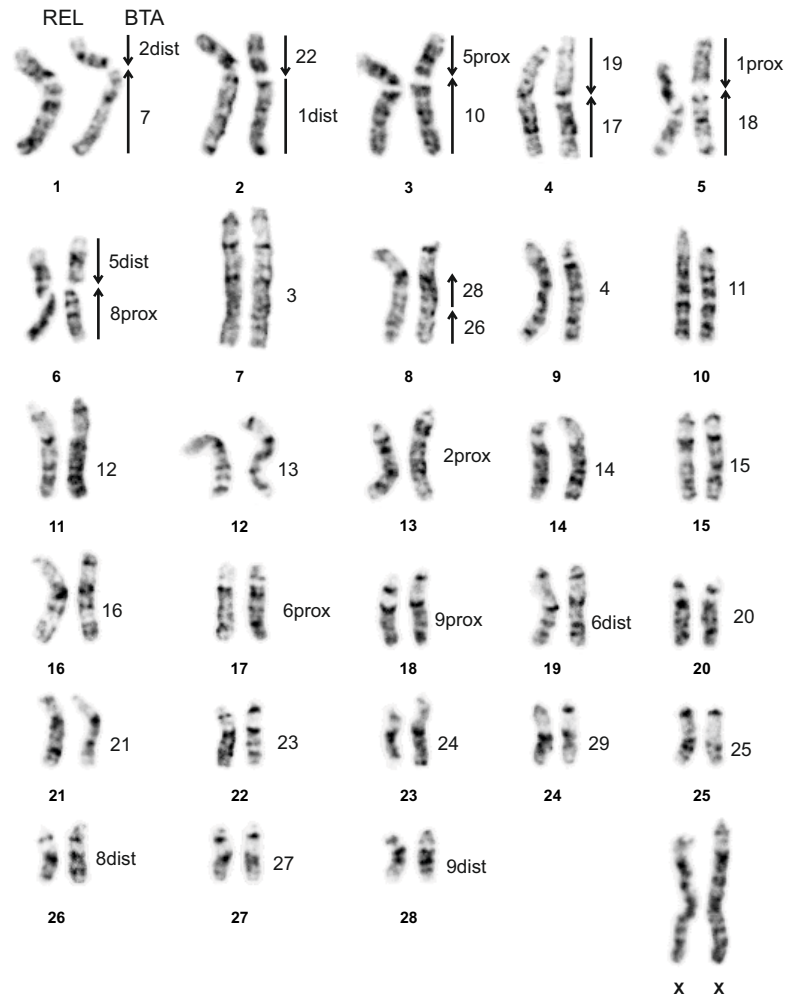

D

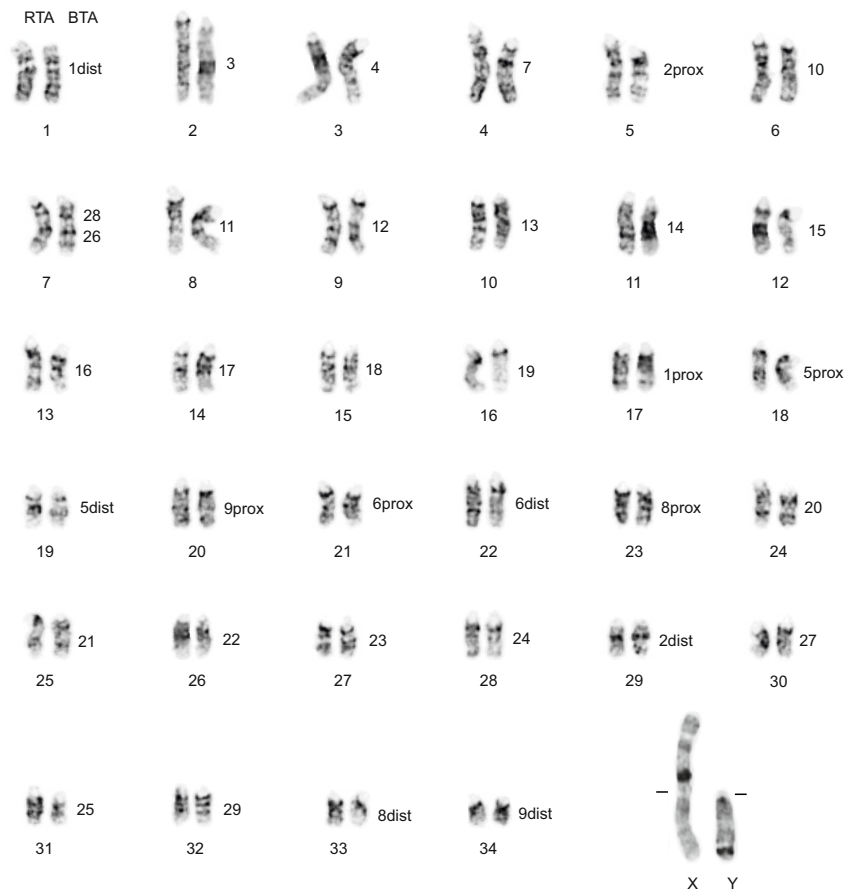

E

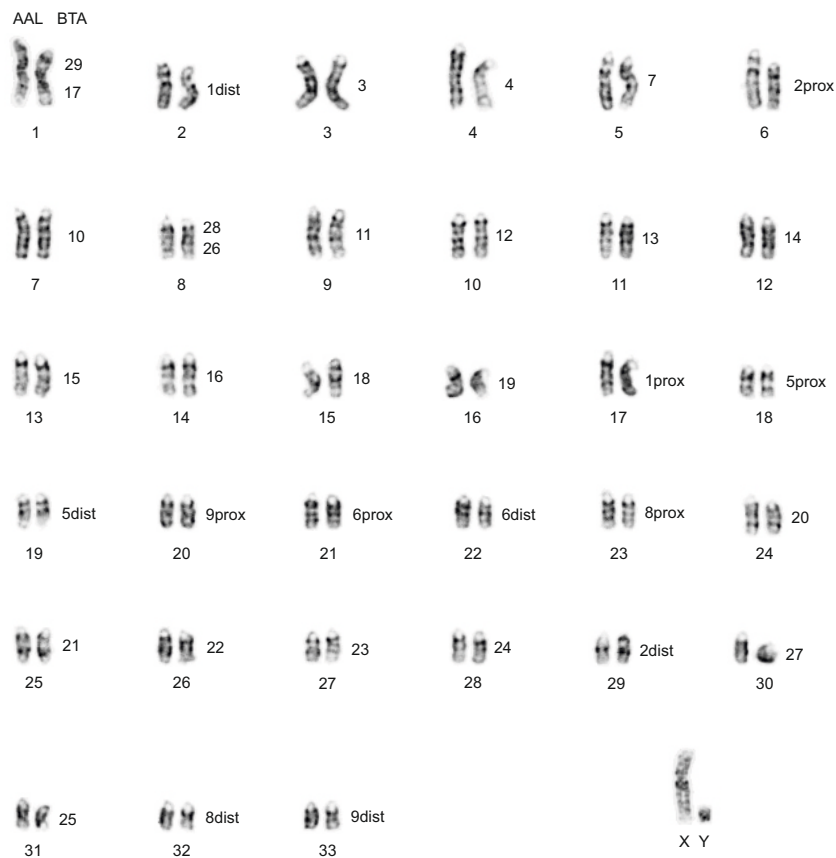

F

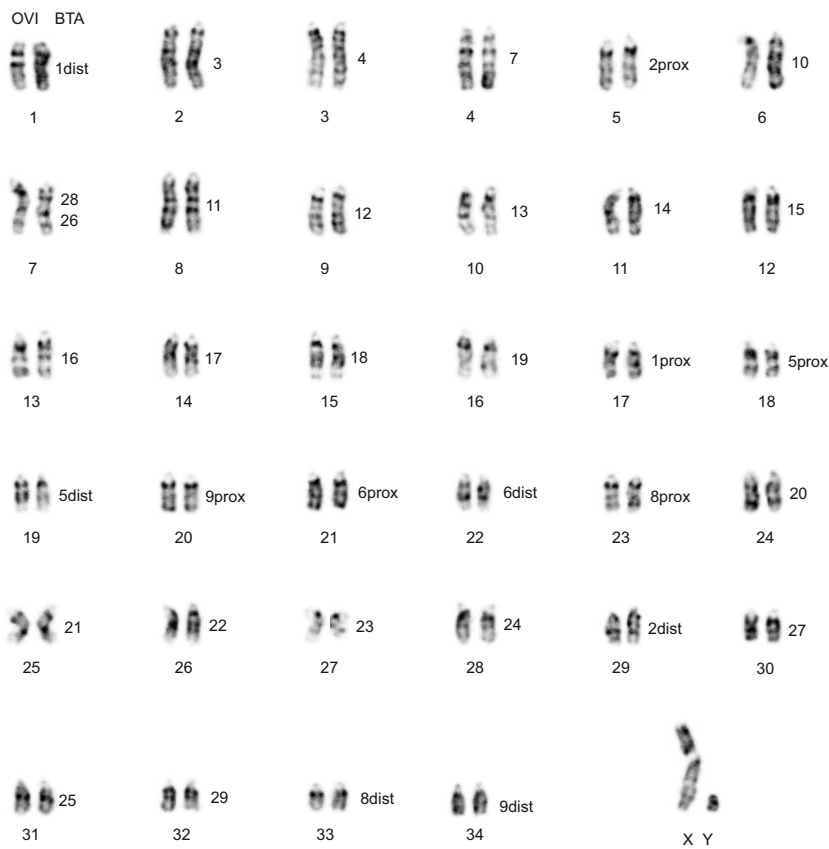

Supplement: Supplementary file 1 [file animals-11-02614-s001.zip › Suppl Figure S2.pdf]

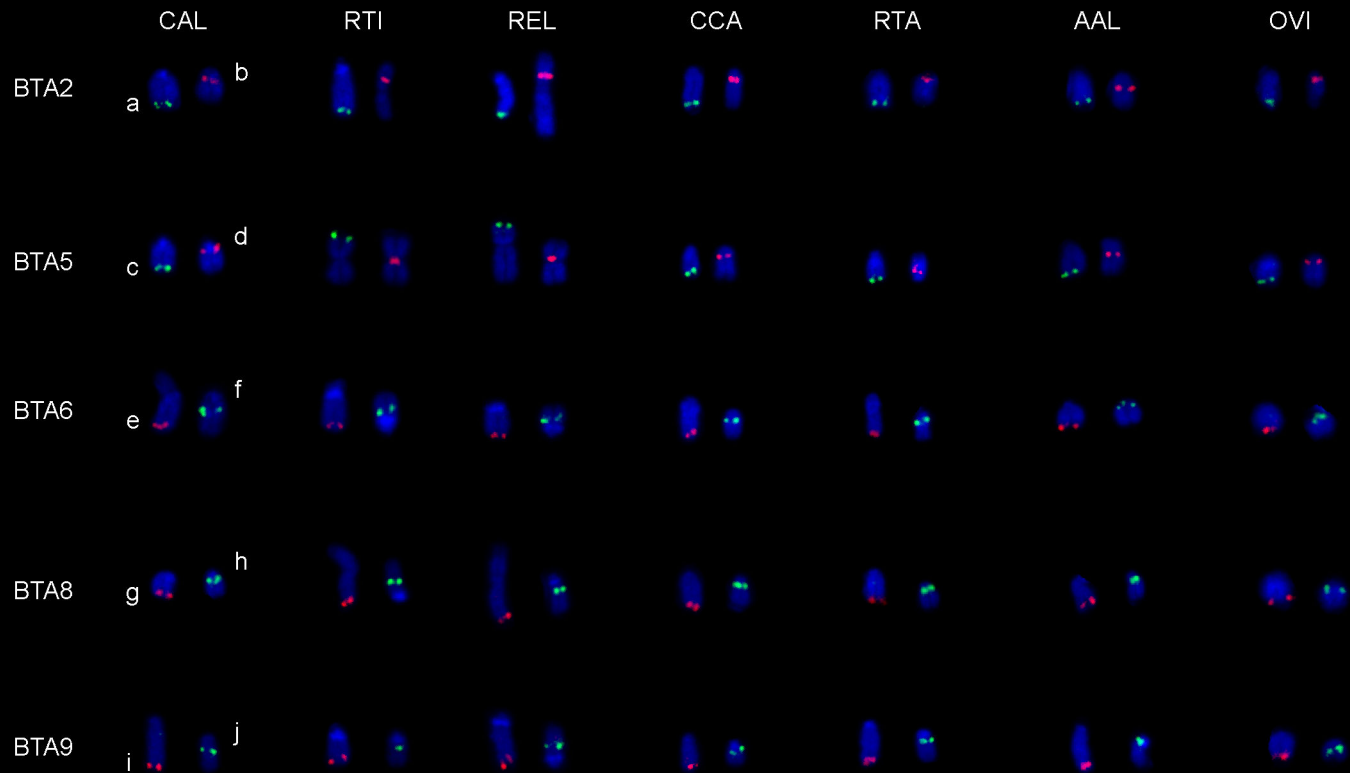

Supplement: Supplementary file 1 [file animals-11-02614-s001.zip › Suppl Figure S3.pdf]
